# Determinants of Vaccine Immunogenicity in HIV-Infected Pregnant Women: Analysis of B and T Cell Responses to Pandemic H1N1 Monovalent Vaccine

**DOI:** 10.1371/journal.pone.0122431

**Published:** 2015-04-13

**Authors:** Adriana Weinberg, Petronella Muresan, Kelly M. Richardson, Terence Fenton, Teresa Dominguez, Anthony Bloom, D. Heather Watts, Mark J. Abzug, Sharon A. Nachman, Myron J. Levin, for the P1086 team

**Affiliations:** 1 University of Colorado Anschutz Medical Center, Aurora, Colorado, United States of America; 2 Statistical and Data Analysis Center, Center for Biostatistics in AIDS Research, Harvard School of Public Health, Boston, Massachusetts, United States of America; 3 Frontier Science and Technology Research Foundation, Buffalo, New York, United States of America; 4 Maternal and Pediatric Infectious Disease Branch, Eunice Kennedy Shriver National Institute of Child Health and Human Development, Bethesda, Maryland, United States of America; 5 State University of New York Health Science Center at Stony Brook, Stony Brook, New York, United States of America; University of Pittsburgh Center for Vaccine Research, UNITED STATES

## Abstract

Influenza infections have high frequency and morbidity in HIV-infected pregnant women, underscoring the importance of vaccine-conferred protection. To identify the factors that determine vaccine immunogenicity in this group, we characterized the relationship of B- and T-cell responses to pandemic H1N1 (pH1N1) vaccine with HIV-associated immunologic and virologic characteristics.

pH1N1 and seasonal-H1N1 (sH1N1) antibodies were measured in 119 HIV-infected pregnant women after two double-strength pH1N1 vaccine doses. pH1N1-IgG and IgA B-cell FluoroSpot, pH1N1- and sH1N1-interferon γ (IFNγ) and granzyme B (GrB) T-cell FluoroSpot, and flow cytometric characterization of B- and T-cell subsets were performed in 57 subjects.

pH1N1-antibodies increased after vaccination, but less than previously described in healthy adults. pH1N1-IgG memory B cells (Bmem) increased, IFNγ-effector T-cells (Teff) decreased, and IgA Bmem and GrB Teff did not change. pH1N1-antibodies and Teff were significantly correlated with each other and with sH1N1-HAI and Teff, respectively, before and after vaccination. pH1N1-antibody responses to the vaccine significantly increased with high proportions of CD4+, low CD8+ and low CD8+HLADR+CD38+ activated (Tact) cells. pH1N1-IgG Bmem responses increased with high proportions of CD19+CD27+CD21- activated B cells (Bact), high CD8+CD39+ regulatory T cells (Treg), and low CD19+CD27-CD21- exhausted B cells (Bexhaust). IFNγ-Teff responses increased with low HIV plasma RNA, CD8+HLADR+CD38+ Tact, CD4+FoxP3+ Treg and CD19+IL10+ Breg.

In conclusion, pre-existing antibody and Teff responses to sH1N1 were associated with increased responses to pH1N1 vaccination in HIV-infected pregnant women suggesting an important role for heterosubtypic immunologic memory. High CD4+% T cells were associated with increased, whereas high HIV replication, Tact and Bexhaust were associated with decreased vaccine immunogenicity. High Treg increased antibody responses but decreased Teff responses to the vaccine. The proportions of immature and transitional B cells did not affect the responses to vaccine. Increased Bact were associated with high Bmem responses to the vaccine.

## Introduction

Pregnant women in their 2^nd^ and 3^rd^ trimesters and the first 2 weeks post-partum have a 3.3- to 5.5-fold greater risk of hospitalization for influenza-associated acute cardio-respiratory illness compared to non-pregnant women[1–6]. Additionally, influenza respiratory illness during pregnancy may increase the risk of premature delivery, fetal distress and emergency caesarean sections[7,8]. Heightened susceptibility to severe influenza illness during pregnancy is particularly evident during influenza pandemics as was observed during the pandemic caused by the pandemic influenza A H1N1 2009 (pH1N1)[1–3,5]. Vaccination is the most effective modality to combat the morbidity of influenza infections[9,10]. Administration of seasonal trivalent inactivated vaccines (IIV3) to pregnant women prevents severe infections in women and their infants up to 6 months of life and decreases premature deliveries[10–16]. Although early studies showed that IIV3 had similar immunogenicity in pregnant women and non-pregnant adults[17], this concept was recently challenged[18,19].

HIV-infected adults do not seem to have greater influenza-associated morbidity than same-age uninfected controls except for those with CD4+ cells <200 cells/μL[20–26]. This conclusion is uncertain with respect to HIV-infected pregnant women in whom the immunosuppressive effect of pregnancy may synergize with that of HIV infection. Furthermore, the immunogenicity of influenza vaccines is much lower in HIV-infected individuals compared with uninfected controls of the same age. We previously showed that HIV-infected pregnant women had lower hemagglutination inhibition (HAI) antibodies and cell-mediated immunity (CMI) in response to IIV3 compared with uninfected pregnant women[27]. Since low CD4+ cell numbers have been associated with poor responses to vaccines in HIV-infected individuals [28–37], it is noteworthy that HIV-infected pregnant women experience a decrease of approximately 100 CD4+ cells/μL during pregnancy. This is particularly relevant, since the efficacy of IIV is predicated on its ability to generate HAI titers ≥1:40. This was based on the early observation that healthy young adults with HAI titers ≥1:40 had a 50% decrease in influenza disease[38]. Although this immune correlate with protection has recently been challenged[39], it continues to be used as a benchmark for evaluating the immunogenicity of influenza vaccines. Currently, the immune correlates of protection against influenza infection in HIV-infected individuals are not known and the mechanisms responsible for their poor antibody responses to IIV are also not well understood.

Antibody responses to influenza vaccines are T-cell dependent and, therefore, are affected by the functionality of T helper 1 (Th1) [40] and T follicular helper (Tfh) cells [41]. Both Th1 and Tfh functions are severely compromised in HIV-infected individuals and may contribute to the low immunogenicity of influenza vaccines [42–44]. In addition, multiple B-cell abnormalities have been identified in HIV-infected individuals [45], which may also play a role in the poor antibody responses to vaccines. Although HIV does not replicate in B cells, it interferes with B-cell function through multiple interactions: gp120 with cellular DC-SIGN; CD40L incorporated into the virion membrane with cellular CD40; and complement fixing HIV antigen-antibody complexes with cellular CD21 [46–52]. In addition, HIV Nef protein can be delivered to the B cells through immunologic synapses with CD4+ T cells and/or macrophages and impede the NFkB pathway, while also activating the suppressor of cytokine signaling (SOCS) pathway [49]. Additional indirect effects of HIV on B cells result from inflammation and lymphopenia. These ultimately translate into impaired immunoglobulin class switch recombination, loss of resting memory B cells (CD21+CD27+), abnormally high proportions of immature (CD10+) and activated (CD21-CD27+, CD95+ and/or CD38+) B cells, and increased B-cell turn-over and apoptosis [49,53–55]. All these factors may contribute to the decreased antibody responses to vaccines [48,56–59]. Furthermore, only some of the B-cell abnormalities are averted by lack of disease progression or reversed with HAART [60,61]. To effectively target efforts to improve vaccine responses in HIV-infected individuals, it is important to understand the relative contributions of each of these factors to decreased antibody responses to vaccines.

The goal of this study was to identify the factors associated with the immunogenicity of IIV in HIV-infected pregnant women. We previously reported that HIV-infected pregnant women enrolled in the P1086 study of the International Maternal Pediatric and Adolescent AIDS Clinical Trials group (IMPAACT) who received two 30μg-doses of pH1N1 monovalent vaccine (IIV1) had lower antibody responses than uninfected historical controls after a single 15μg-dose of the vaccine[62]. Here we evaluated the primary and anamnestic HAI, effector T-cell (Teff) and memory B-cell (Bmem) responses to pH1N1 IIV1 among P1086 participants and the relationships between these endpoints with B- and T-cell phenotypic characteristics. The relationship between immune responses to pH1N1 vaccine and pre-existing immunity to seasonal H1N1 (sH1N1) virus was also studied.

## Materials and Methods

### Study design

P1086 involved human subject research and was approved by the Colorado Multiple Institutional Review Board on 9/25/09; approval number 09–0803. Informed written consent was obtained from all participants. HIV-infected women 18 to 39 years of age, 14 to 34 weeks gestation, and on antiretroviral therapy, received two 30 μg doses of unadjuvanted, inactivated pH1N1 vaccine 21 to 28 days apart at 31 U.S. IMPAACT sites as previously described[62]. Serum was collected at entry, before administration of the 1^st^ dose of vaccine; before administration of the 2^nd^ dose (21 to 28 days post-dose 1); and 10 to 14 days post-dose 2. The 21 to 28 days post-dose 1 and 10 to 14 days post-dose 2 time points were selected to coincide with the anticipated peak primary and anamnestic antibody responses, respectively. Peripheral blood mononuclear cells (PBMCs) were isolated and cryopreserved for viability at the same time points.

### Antibody responses measured by HAI

pH1N1 and sH1N1 HAI titers were measured as previously described [13] using A/California/7/2009 Pandemic X-179A and A/South Dakota/6/2007 H1N1 influenza viruses, respectively, obtained through a generous gift of Dr. A Klimov from the Centers of Disease Control. HAI titers were expressed as the reciprocal of the endpoint titer. Seroprotection was defined as a titer ≥ 40.

### IFNγ and Granzyme B (GrB) CMI assays

PBMCs were cryopreserved according to a standardized protocol (http://www.hanc.info/labs/Pages/SOPs.aspx) at the local laboratories, which were previously certified for PBMC cryopreservation; stored at ≤-150°C; and shipped in liquid nitrogen dry shippers to the testing lab at University of Colorado Anschutz Medical Campus. Cells were thawed slowly as previously described.[63] FluoroSpot assays were performed using commercial dual color IFNγ/Granzyme B FluoroSpot kits (MabTech) per manufacturer’s instructions with modifications. Thawed PBMCs were maintained overnight at 10^6^ PBMC/mL in RPMI 1640 with glutamine (Gibco), 10% human AB serum (Gibco), 1% penicillin and streptomycin, and 1% Hepes buffer (Corning Cellgrow) at 37°C in a humidified 5% CO_2_ atmosphere. PBMCs with viability <70%, as measured with a Guava easyCyte 8HT instrument (Millipore), were excluded from the subsequent steps to avoid biasing the results by decreased viability[63–65]. PBMCs at 250,000 cells/well were stimulated in duplicate wells with 2 TCID_50_/PBMC of A/California/7/2009 Pandemic X-179A and A/South Dakota/6/2007 H1N1 influenza viruses, 5 μg/mL phytohemagglutinin (PHA; Sigma) or medium control. After a 48-h incubation at 37°C in a humidified, 5% CO_2_ atmosphere, plates were washed and stained with anti-IFNγ and GrB mAbs as per manufacturer’s instructions. Spot forming cells (SFCs) were counted with an ImmunoSpot II Analyzer (Cell Technologies Ltd). Results were expressed as SFC/10^6^ PBMC after subtracting the SFC in unstimulated control wells from those enumerated in antigen- or mitogen- stimulated wells.

### IgG and IgA FluoroSpot

Cryopreserved PBMCs were used for the detection of IgG and IgA antibody secreting cells (ASC). PBMCs were thawed, counted and then stimulated in RPMI 1640 (Gibco) with 10% fetal bovine serum (Gemini Bio-Products), 0.4% penicillin and streptomycin, 1% Hepes buffer (Corning Cellgrow), 1μg/mL *Staphylococcus aureus* Cowan (Sigma-Aldrich), 6μg/mL CpG (Operon Technologies), 100ng/mL recombinant human IL-10 (Cell Sciences), and 1μg/mL pokeweed mitogen (Sigma-Aldrich) for 96 hours at 37° C, 5% CO_2_, which proved to be optimal conditions in our optimization assays. Stimulated PBMCs were used at 50,000 cells/well in duplicate wells of the FluoroSpot IgG/IgA kits (Mabtech Inc.). Assays were performed as per manufacturer’s instructions in plates coated with pH1N1 antigen at 4.1 HA units/well. ASC were enumerated using the ImmunoSpot II instrument. Results were expressed as ASC/10^6^ stimulated PBMCs.

### B- and T-cell phenotypic characterization

B- and T-cell subsets were enumerated in freshly thawed cryopreserved PBMCs. After washing and counting viable cells, PBMCs were surface-stained with the following conjugated mAbs: anti-CD3-AF488 (Biolegend; clone HIT3a), anti CD8-APC/Cy7 (Biolegend; SK1), anti-CD19-APC/Cy7 (BD Biosciences; SJ25C1), anti-CD19-PerCP/Cy5.5 (Biolegend; HiB19) anti-CD25-PE/Cy7 (Biolegend; BC96), anti-CD21-PE/Cy7 (BD Biosciences; B-ly4), anti-CD27-PerCP/Cy5.5 (BD Biosciences; M-T271), anti-CD38-PE/Cy7 (Biolegend; HIT2), anti-HLA-DR-PerCP/Cy5.5 (Biolegend; L243); anti-CD10-FITC (BD Biosciences; W8E7), anti-IL21R-PE (BD Biosciences; 17A12), and anti-CD39-APC (Biolegend; A1). Cells were fixed and permeabilized with Cytofix/Cytoperm (BD Biosciences), and stained with anti-IL-10-PE (R & D Systems; 127107), anti-FOXP3-PE (Biolegend; 206D) and anti- TGFβ-PE (Biolegend; TW4-2F8), and analyzed with Guava easyCyte 8HT and FlowJo (Treestar). Subsets were expressed as percentages of the parent CD4+, CD8+ and CD19+ cell populations. The gating strategy is presented in S2 Fig.

### Statistical methods

This substudy was an exploratory project and no sample size calculations were performed.* Its objectives included descriptive and correlation analyses, which did not require sample size estimation to generate interpretable results. HAI titers <10 were considered undetectable and were assigned a value of 5. Changes in pH1N1 and sH1N1 HAI titers, T and B-cell mediated immune responses, and phenotypic characterization of T and B-cell subsets from baseline to post immunization or from post-dose 1 to post-dose 2 were assessed using Wilcoxon Matched Pairs Signed-Rank tests. The final flow cytometric analyses were restricted to samples with ≥100 events in the anchor gate. However, a sensitivity analysis showed that the inclusion of samples with <100 events in the anchor gate would not have changed the results. Spearman correlation analyses were performed to assess the strength of associations and test their statistical significance. All analyses were performed using SAS Version 9.2 (SAS Institute INC, Cary, NC) and the graphs were produced using the R software.

## Results

### Demographic and HIV Disease Characteristics of the Study Population

Of the 119 women on HAART at the time of immunization in the parent study, who received both immunizations prior to delivery and had antibodies measured, 57 had complete sets of PBMCs with adequate viability for the CMI substudy, although in some cases there were not enough cells to perform all CMI assays. The demographic and HIV disease characteristics of the CMI substudy participants did not differ from those of the entire cohort (Table 1). Of the 119 participants, 36 received IIV3 ≥14 days before study entry and 5 received it ≥21 days after the 2^nd^ dose of the pH1N1 IIV1.

**Table 1:** Characteristics of the study population.

Characteristic	All	Substudy
Number of Subjects	119	57
Race and Ethnicity		
Black	71 (60%)	32 (56%)
Latino	41 (34%)	25 (44%)
Age (yrs)		
Median	29	27
Inter Quartile Range (IQR)	(22,32)	(22,30)
Gestational Age (wks)		
Median	25	26
QR	(20,29)	(20,30)
cART	119 (100%)	57 (100%)
Type of ARV Regimen		
HAART	112 (94%)	51 (89%)
Other	7 (6%)	6 (11%)
CD4 Percent		
Median	32	32
IQR	(23,39)	(27,41)
CD8 Percent		
Median	46	46
IQR	(38,52)	(38,52)
Log 10 RNA Count^a^		
Median	1.9	2.0
IQR	(1.7,2.6)	(1.7,2.6)
HIV RNA < 50 cp/mL	42 (35%)	18 (32%)

^a^ The lower limit of detection varied among subjects depending on the assay used at the clinical research site; RNA values below the limit of detection were replaced with the lower detection limit of the assay.

### pH1N1- and sH1N1-Immune Responses and Their Inter-Relationships

#### Antibodies

**Fig 1:**
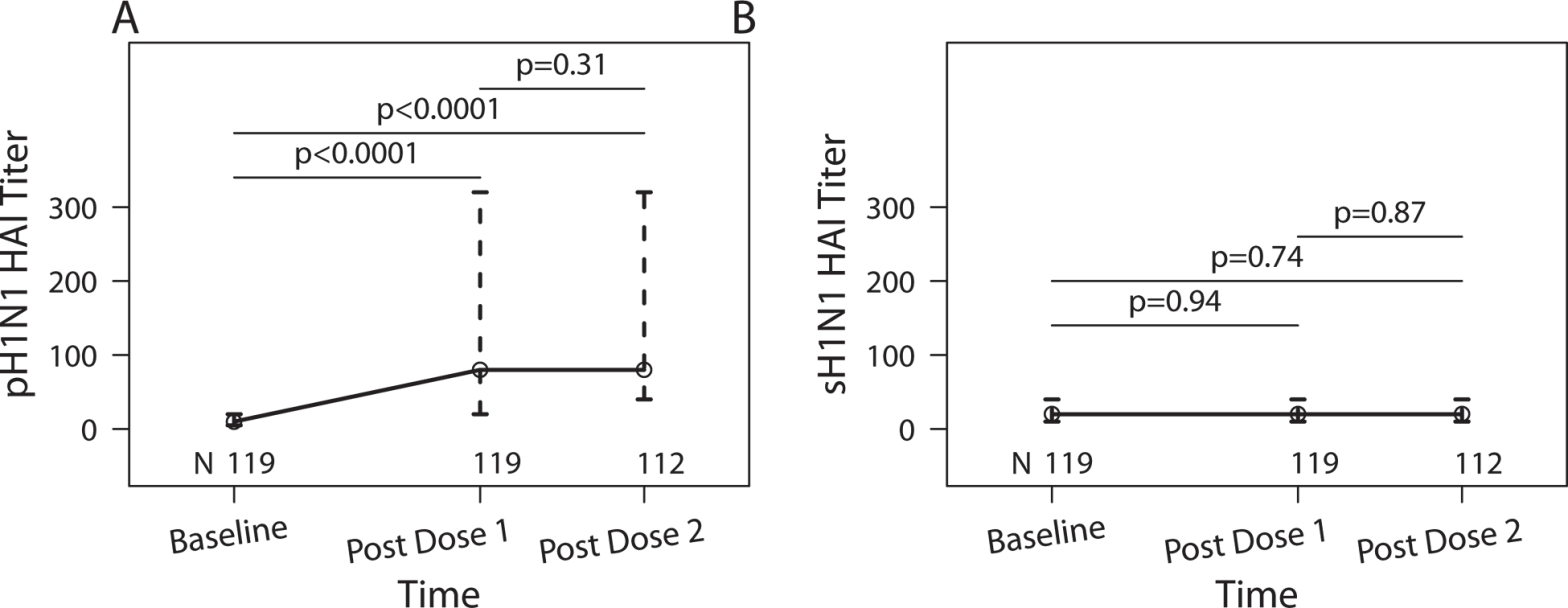
pH1N1 and sH1N1 HAI titers of HIV-infected pregnant women who received two doses of pH1N1 monovalent vaccine. Data represent medians and inter quartile ranges of the reciprocals of the HAI titers. The number of subjects who contributed data and p values of paired comparisons are indicated on the graph. Panel A shows pH1N1 antibody titers and panel B shows sH1N1 antibody titers.

Fig 1 shows HAI antibody titers against pH1N1 and sH1N1 over time in participants who received both doses of pH1N1 vaccine before delivery (consort diagram in S1 Fig). At baseline, the medians of the reciprocal of the HAI titers for pH1N1 and sH1N1 were 10 and 20, respectively. pH1N1 titers significantly increased post-dose 1 of vaccine (median = 80, p<0.001; Fig 1A), but sH1N1 titers did not (p = 0.94; Fig 1B). There were no significant differences in pH1N1 or sH1N1 antibody titers between post-doses 1 and 2 (p = 0.31 and 0.87, respectively).

#### Memory B cells (Bmem)

**Fig 2:**
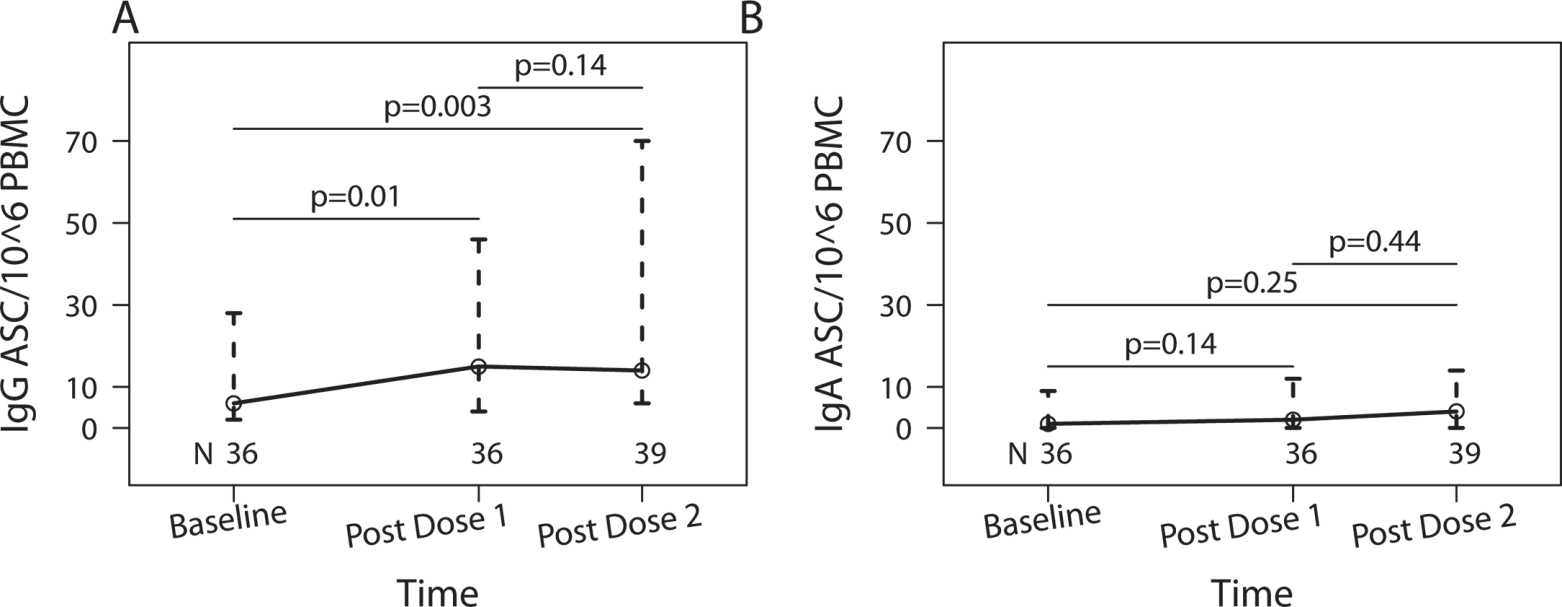
pH1N1 IgG and IgA B-cell memory responses of HIV-infected pregnant women who received two doses of pH1N1 monovalent vaccine. Data represent medians and inter quartile ranges of antibody secreting cells (ASC)/10^6^ PBMC. The number of subjects who had adequate numbers of viable PBMC to complete the assays at each time point is indicated on the graph. p values calculated with Wilcoxon Matched Pairs Signed-Rank test are indicated on the graph. Panel A shows IgG ASC/10^6^ PBMC and panel B shows IgA ASC/10^6^ PBMC.

Fig 2 shows the kinetics of pH1N1-specific Bmem IgG and IgA ASC measured by FluoroSpot. There was a significant increase in IgG ASCs post-dose 1 (from median of 6 to 15 ASC/10^6^ PBMC; p = 0.01; Fig 2A), but no further increases post-dose 2 (median = 14 ASC/10^6^ PBMC; p = 0.14) compared to post-dose 1. IgA ASCs were barely detectable at baseline (median = 1 ASC/10^6^ PBMC) and did not significantly increase after vaccination (Fig 2B).

#### Effector T cells (Teff)

**Fig 3:**
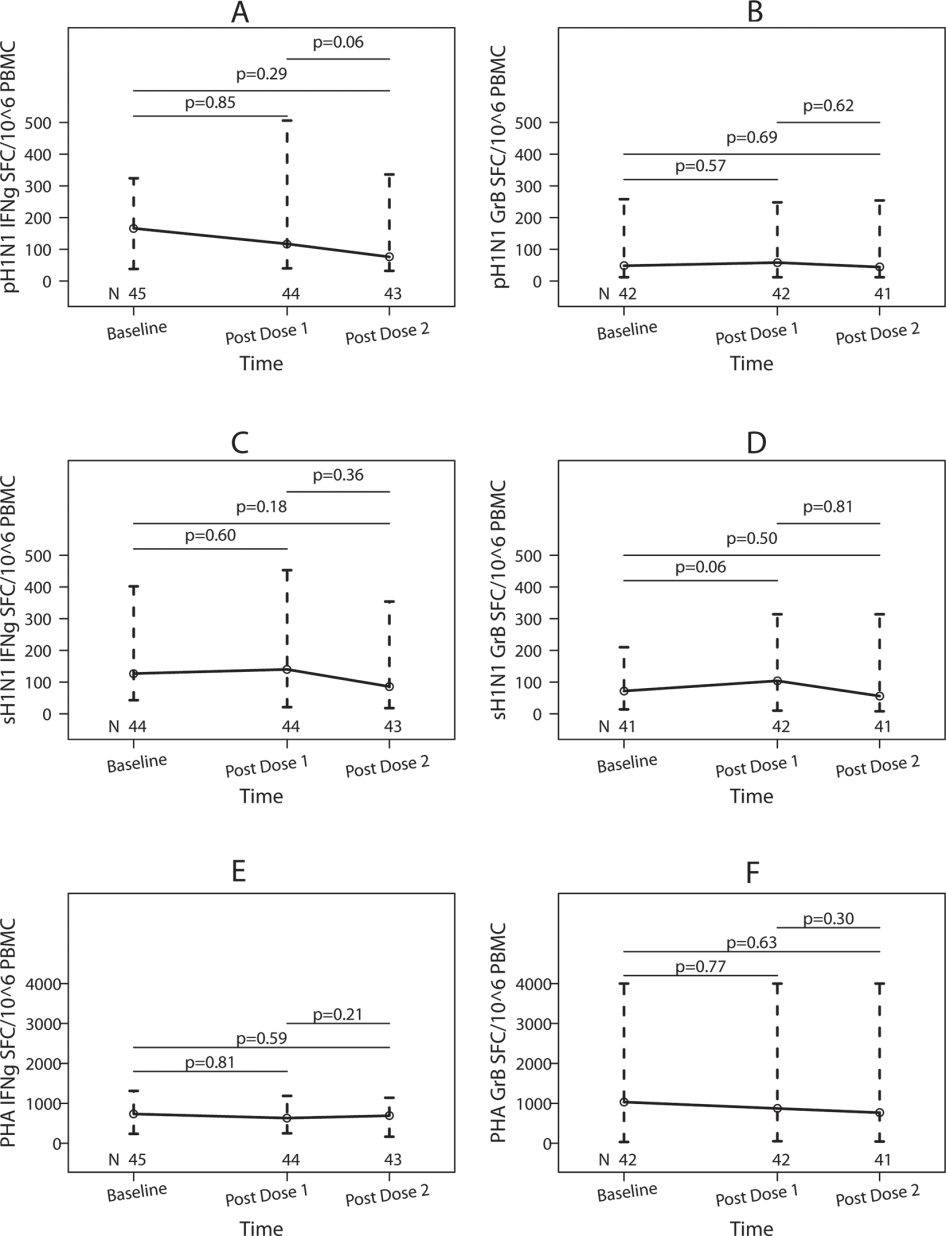
pH1N1 and sH1N1 effector T cell responses of HIV-infected pregnant women who received two doses of pH1N1 monovalent vaccine. Data represent medians and inter quartile ranges of spot forming cells (SFC)/10^6^ PBMC. The number of subjects who had adequate numbers of viable PBMC to complete the assays at each time point is indicated on the graph. p values calculated with Wilcoxon Matched Pairs Signed-Rank test are indicated on the graph. Panel A shows pH1N1-IFNγ SFC/10^6^ PBMC, panel B pH1N1-granzyme B (GrB) SFC/10^6^ PBMC, panel C sH1N1-IFNγ SFC/10^6^ PBMC, panel D sH1N1-GrB SFC/10^6^ PBMC, panel E PHA-IFNγ SFC/10^6^ PBMC positive controls, and panel F PHA-GrB SFC/10^6^ PBMC controls.

pH1N1- and sH1N1-specific Teff were measured by IFNγ and GrB FluoroSpot (Fig 3). Appreciable pH1N1-IFNγ Teff were present at baseline (median = 166 SFC/10^6^ PBMC; Fig 3A) and tended to decrease after vaccination, particularly at post-dose 2 (median = 76 SFC/10^6^ PBMC). In contrast, pH1N1-GrB responses were modest at baseline (median = 48 SFC/10^6^ PBMC; Fig 3B) and did not appreciably change after vaccination. sH1N1 FluoroSpot revealed no significant changes in IFNγ SFCs after either dose of vaccine (Fig 3C), but exhibited a marginally significant increase in GrB SFCs post-dose 1 (p = 0.06; Fig 3D). There were no significant changes in PHA-stimulated IFNγ (Fig 3E) or GrB (Fig 3F) SFCs during the study.

#### Associations of humoral and cellular pH1N1- and sH1N1-specific responses in pH1N1 IIV1 recipients

As shown in Table 2, there were significant associations between pH1N1- and sH1N1-HAI titers at all time points. In the subset of women who had Bmem and Teff measured, pH1N1-HAI titers and pH1N1-IFNγ SFCs were significantly correlated at baseline (rho = 0.35; p = 0.02) and post-dose 1 (rho = 0.32; p = 0.04), but only marginally correlated at post-dose 2 (rho = 0.29; p = 0.06). pH1N1-IFNγ and pH1N1-GrB SFCs were highly correlated with each other and with sH1N1-IFNγ and sH1N1-GrB SFCs at all time points (rho≥0.65; p<0.0001). pH1N1 IgG ASCs correlated with pH1N1-IgA ASCs, but not with any other influenza-specific responses.

**Table 2:** Selected correlations of cellular and humoral B and T cell-mediated immune responses to pH1N1 and sH1N1.

Variable 1	Variable 2	Baseline		Post-dose 1		Post-dose 2	
		ρ* (p value)	N	ρ* (p value)	N	ρ* (p value)	N
*pH1N1 HAI Titers*	sH1N1 HAI	0.25 (0.01)	119	0.27 (0.003)	119	0.24 (0.01)	112
	pH1N1 IgG ASC	-0.11 (0.54)	36	-0.05 (0.77)	36	0.07 (0.68)	39
	pH1N1 IFNγ SFC	0.35 (0.02)	45	0.32 (0.04)	44	0.29 (0.06)	43
*pH1N1 IgG ASC*	sH1N1 HAI	-0.13 (0.46)	36	-0.08 (0.62)	36	0.03 (0.86)	39
	pH1N1 IgA ASC	0.65 (<0.0001)	36	0.59 (0.0002)	36	0.50 (0.001)	39
	pH1N1 IFNγ SFC	0.07 (0.71)	29	-0.12 (0.52)	31	0.15 (0.42)	32
*pH1N1 IFN*γ *SFC*	sH1N1 HAI	-0.12 (0.43)	45	0.05 (0.76)	44	0.11 (0.47)	43
	pH1N1 GrB SFC	0.68 (<0.0001)	42	0.78 (<0.0001)	42	0.67 (<0.0001)	41
	sH1N1 IFNγ SFC	0.92 (<0.0001)	44	0.88 (<0.0001)	44	0.85 (<0.0001)	43
	sH1N1 GrB SFC	0.65 (<0.0001)	41	0.74 (<0.0001)	42	0.72 (<0.0001)	41

*Spearman correlation coefficients. Dark shading represents the values that were significant at a 0.05 level. Lighter shading indicates the values that were marginally significant at 0.05–0.1.

### Effect of HIV disease characteristics on immune responses to pH1N1 vaccine

#### CD4, CD8 and HIV plasma RNA

Correlation analyses of pH1N1 immune responses with CD4%, CD8% and HIV plasma RNA (Table 3) showed that HAI titers after vaccination significantly increased with higher CD4% and with low CD8%, both post-dose 1 and post-dose 2 (CD4%: rho≥0.32, p≤0.001; CD8%: rho≤-0.24, p≤0.02). There were no significant correlations of HIV plasma RNA with pH1N1-HAI titers after vaccination. In the subset of women who had Bmem and Teff measured, neither IgG nor IgA ASC correlated with CD4%, CD8% or plasma HIV RNA at any time points. However, IFNγ and GrB SFC showed significant negative correlations with HIV plasma RNA at all time points (rho≤-0.37, p≤0.01).

**Table 3:** Correlations of pH1N1 immune responses with plasma HIV RNA, CD4% and CD8%.

Variable 1	Variable 2 at baseline	Post-dose 1		Post-dose 2	
		ρ* (p value)	N	ρ* (p value)	N
*HAI Titers*	CD4%	0.34 (0.0001)	119	0.32 (0.001)	112
	CD8%	-0.30 (0.001)	119	-0.30 (0.02)	112
	HIV RNA cp/mL	-0.11 (0.25)	119	-0.11 (0.26)	112
*IgG ASC*	CD4%	-0.003 (0.99)	36	0.12 (0.45)	39
	CD8%	-0.09 (0.62)	36	-0.03 (0.85)	39
	HIV RNA cp/mL	-0.11 (0.53)	36	-0.14 (0.39)	39
*IgA ASC*	CD4%	-0.02 (0.92)	36	0.01 (0.93)	39
	CD8%	-0.31 (0.07)	36	-0.11 (0.49)	39
	HIV RNA cp/mL	-0.14 (0.42)	36	-0.14 (0.40)	39
*IFN*γ *SFC*	CD4%	0.08 (0.62)	44	0.28 (0.07)	43
	CD8%	-0.04 (0.81)	44	-0.27 (0.08)	43
	HIV RNA cp/mL	-0.37 (0.01)	44	-0.43 (0.004)	43
*GrB SFC*	CD4%	-0.01 (0.94)	42	0.09 (0.57)	41
	CD8%	0.07 (0.66)	42	-0.13 (0.43)	41
	HIV RNA cp/mL	-0.43 (0.01)	42	-0.46 (0.003)	41

#### Activated, regulatory, immature and exhausted T and B cell subsets

To determine whether increased activation or regulation, characteristic of HIV disease, or other HIV-associated abnormalities of lymphocyte subsets played a role in the responses to pH1N1 IIV1, we measured T and B cell subsets representative of activation and regulation and B cell subsets that are typically skewed by HIV infection and may play important roles in antibody responses to vaccines. The following subsets were measured at baseline and after each dose of vaccine: CD19+CD10+, CD19+IL-21R+, CD19+CD27+CD10+ = activated immature B cells, CD19+CD27-CD10+ = immature B cells, CD19+CD27-CD21+ = naïve B cells, CD19+CD27+CD21+ = resting memory B cells, CD19+CD27+CD21- = transitional B cells, CD19+CD27-CD21- = exhausted B cells, CD4+CD39+ = regulatory T cells, CD4+HLADR+CD38+ = activated T cells, CD4+ TGFβ+ = regulatory T cells, CD8+CD39+ = regulatory T cells, CD8+HLADR+CD38+ = activated T cells, CD8+ TGFβ+ = regulatory T cells, CD4+IL10+ = regulatory T cells, CD4+FOXP3+ = regulatory T cells, CD4+CD25+FOXP3+ = regulatory T cells, CD8+IL10+ = regulatory T cells, CD8+FOXP3+ = regulatory T cells, CD8+CD25+FOXP3+ = regulatory T cells, CD19+IL10+ = regulatory B cells and CD19+CD25+ = regulatory B cells. There were no significant changes from baseline to the post-dose 1 and post-dose 2 time points in any of the T or B cell subsets measured (S1 Table).

Correlation analyses of the pH1N1-specific humoral and cellular immune responses with the above listed lymphocyte subsets showed several significant associations (Table 4). HAI antibody responses decreased with high CD8+HLADR+CD38+% activated T cells at all time points (rho≤-0.37, p≤0.02) and marginally increased with CD8+IL10+% regulatory T cells at post-dose 2 (rho = 0.30, p = 0.05). IgG ASC decreased with high CD19+CD27-CD21-% exhausted B cells at all time points (rho≤-0.36, p≤0.03); IgG ASC increased with high CD8+CD39+% regulatory T cells (rho = 0.42, p = 0.02) at post-dose 1 and with high CD19+CD27+CD21-% activated B cells (rho = 0.35, p = 0.04) at post-dose 2. IFNγ SFCs inversely correlated with: CD8+HLADR+CD38+% activated T cells at post-dose 1 (rho = -0.40, p = 0.03) and with CD19+IL10+% regulatory B cells (rho = -0.36, p = 0.047) and marginally with CD4+TGF (rho = -.33, p = 0.07) at post-dose 2.

**Table 4:** Correlations of pH1N1 immune responses with B- and T-Cell subsets.

Variable 1	Variable 2 (%)^a^	Post-dose 1		Post-dose 2	
		ρ* (p value)	N	ρ* (p value)	N
*HAI Titers*	CD8+HLADR+CD38+	-0.48 (0.002)	40	-0.37 (0.02)	42
	CD8+IL10+	0.20 (0.22)	42	0.30 (0.05)	43
*IgG ASC*	CD19+CD27+CD21-	0.14 (0.44)	35	0.35 (0.04)	36
	CD19+CD27-CD21-	-0.36 (0.03)	35	-0.50 (0.002)	36
	CD8+CD39+	0.42 (0.02)	33	-0.04 (0.82)	36
*IFN*γ *SFC*	CD4+ TGF+	-0.15 (0.43)	29	-0.33 (0.07)	31
	CD8+CD39+	-0.34 (0.07)	29	-0.07 (0.70)	31
	CD8+HLADR+CD38+	-0.40 (0.03)	29	-0.05 (0.78)	31
	CD4+IL10+	-0.02 (0.91)	31	-0.31 (0.09)	31
	CD19+IL10+	-0.12 (0.52)	31	-0.36 (0.047)	31

## Discussion

This study identified HIV disease characteristics other than CD4 or CD8 cell numbers and HIV viral load that may modulate immune responses to pH1N1 IIV1 in HIV-infected pregnant women. The proportions of activated and regulatory B and T cells and exhausted B cells significantly correlated with pH1N1-specific HAI, Teff, and/or Bmem responses to the vaccine, including some unexpected associations.

The correlation between HAI antibody titers ≥40 and decreased incidence of symptomatic influenza disease suggests that the factors that modulate antibody responses to IIVs are highly relevant for vaccine efficacy. During typical seasonal epidemics, some of the antibody responses are anamnestic, as large proportions of antibodies arise from Bmem[66]. However, during pandemics the primary antibody response is crucial, since adults typically receive a single dose of vaccine to prevent disease. This is especially problematic for HIV-infected patients, who predictably have low antibody responses to influenza vaccines, and for whom there is no recommendation to modify the immunization schedule. In this study, both primary (post-dose 1) and anamnestic (post-dose 2) pH1N1 antibody responses significantly increased with high proportions of CD4 and with low proportions of total CD8 and of CD8 activated T cells, but not with plasma HIV RNA. The proportions of activated T cells in HIV-infected individuals generally mirror viral replication as measured by HIV plasma RNA[67], but in this study, HIV plasma RNA did not correlate with pH1N1-HAI responses. Activated T cells may be elevated even if the plasma viral load is below the limit of detection of conventional HIV RNA PCR assays, and this may be frequently the case shortly after starting cART, which was true for some of the study participants. Other viral and bacterial pathogens, the best known of which is CMV[68], may increase CD8+ activated T cells and may explain the discordance of the effects of CD8+ activated T cells and HIV viral load. A direct mechanism whereby activated T cells suppress de novo immune responses has not been described, however a recent study showed that immune activation decreased immune responses to yellow fever vaccine in individuals without HIV infection[69]. We also previously showed that reconstitution of antigen-specific T-cell responses in HIV-infected children on cART decreased with elevated CD8+ activated T cells[70]. Taken together these data suggest that high frequencies of CD8+ activated T cells may be at least a marker of a dysfunctional immune system and could be used to optimize the timing of vaccine administration to HIV-infected individuals.

Administration of pH1N1 IIV1 generated IgG Bmem after the 1^st^ dose of vaccine without additional increases after the 2^nd^ dose. The magnitude of the pH1N1-Bmem response was similar to that previously described after IIV3 in HIV-infected adults[71,72]. IgA Bmem did not increase after either dose of vaccine, but the magnitude of IgA Bmem significantly correlated with the IgG Bmem at all time points. This is explained by the fact that subjects with high frequencies of IgG Bmem also had relatively high frequencies of IgA Bmem before vaccination and the increase in IgG Bmem after vaccination did not change the rank order of the subject results. The corollary of this observation may be that HIV-infected pregnant women with pre-existing memory to vaccine antigens mount better IgG responses after vaccination than those without pre-existing memory. However, the IgA response is depressed regardless of pre-existing memory. Healthy adults have been previously shown to mount significant IgA Bmem in response to IIV3[71]. The low and heterogeneous IgA Bmem response to IIV1 in our study subjects was likely due to the HIV-associated immune suppression, although an effect of pregnancy cannot be excluded due to the lack of an uninfected control group in this study and the absence of historical information on IgA responses to IIV in pregnant women.

High proportions of exhausted B cells negatively impacted the magnitude of the pH1N1-IgG Bmem responses to vaccination. HIV infection is characterized by an increase in activated B cells, exhausted B cells and immature B-cell subsets. The effect of the change in B-cell subset relative proportions on vaccine responses during pregnancy complicated by HIV infection has not been previously characterized. Although increased proportions of exhausted B cells attenuated the Bmem responses to pH1N1 vaccine, the increased proportions of immature B cells did not affect the IgG Bmem responses. High proportions of activated B cells after the 2^nd^ dose of vaccine were significantly associated with increased pH1N1-IgG Bmem responses, suggesting that vigorous anamnestic Bmem responses to vaccines increase the frequency of circulating activated B cells. cART decreases polyclonal activated B cells and partially corrects the distribution of other B-cell subsets; however, in HIV-infected subjects, even after sustained inhibition of the HIV replication, the resting Bmem subset continues to be lower and the activated, exhausted and immature B cell subsets remain higher compared with HIV-uninfected age-matched controls[54,60] offering a potential explanation for low vaccine immunogenicity in HIV-infected individuals on cART. Although early initiation of cART has a better restorative effect on the B-cell compartment compared with initiation in the chronic phase of HIV infection, recent reports showed a lack of normalization of the B cell subsets even in early-treated individuals[73]. Taken together, these observations suggest that defective Bmem responses to IIV, and perhaps to other inactivated vaccines, may continue as a sustained complication of HIV infection, and that persistence of vaccine-conferred antibody-mediated protection may be different in HIV-infected compared with uninfected individuals. This underscores the importance of studying the persistence of antibodies and the ability to elicit anamnestic responses to vaccines in HIV-infected individuals.

pH1N1-Bmem paradoxically increased after the 1^st^ dose of vaccine with high CD8+CD39+% Tregs. CD39 is an ectonucleosidase that hydrolyzes ATP and ADP[74], and in conjunction with CD73, which is usually co-expressed with CD39, hydrolyzes extracellular ATP to adenosine. This results in a reduction of the pro-inflammatory activity of extracellular ATP and an increase of the anti-inflammatory effect of adenosine. This mechanism is deemed to mediate the regulatory activity of CD39+ regulatory T cells. However, extracellular adenosine also contributes to immunoglobulin class switch[75], which may explain the association found in this study between the frequency of CD8+CD39+ regulatory T cells and pH1N1-IgG Bmem responses to primary immunization. Alternatively, the relationship between IgG Bmem and CD8+CD39+% Treg might not be causational.

The generation of influenza-specific Teff in response to IIV1 is also important because it provides a second line of protection against severe infection. If antibody-mediated protection is insufficient to prevent infection, the Teff become responsible for eliminating influenza-infected cells and limiting viral spread. The second dose of pH1N1 IIV1 resulted in a decrease of pH1N1-specific IFNγ Teff. Although pH1N1-GrB Teff did not change after vaccination, they were highly associated with IFNγ Teff at all time points. The low and heterogeneous GrB Teff responses may reflect the low CD8+ T cell stimulatory ability of IIVs. pH1N1-Teff significantly decreased with high plasma HIV load, activated CD8+ T cells and regulatory B cells and moderately decreased with CD8+CD39+, CD4+TGFβ+ and CD4+IL10+ regulatory T cells. Both HIV infection and pregnancy increase the proportions of regulatory T cells[76–78], but regulatory T cells associated with HIV infection probably play the dominant role in attenuating Teff responses to IIV, since the decrease of Teff after influenza vaccination in previously primed non-pregnant HIV-infected individuals has been a recurrent and consistent observation in our studies [79,80]. CMI decreases were not observed in HIV-uninfected individuals after IIV3[81]. In a previous study, we showed that pH1N1-specific CD8+FOXP3+ and CD8+TGFβ+ regulatory T cells increased after the first dose of pH1N1 IIV1 in HIV-infected children and youth[80]. Although these are not the same regulatory T cell subsets that were found to be associated with decreased pH1N1-IFNγ Teff in this study, this observation suggests that pH1N1 IIV1 administration may lead to an increase in regulatory T cells that may dampen subsequent recall Teff responses. Further studies are needed to determine the fine specificity of circulating regulatory T cells after IIV administration and their ability to block the expansion of influenza-specific Teff.

It is important to note the association of pH1N1 and sH1N1 antibody and Teff responses at all time points. pH1N1 and the immediately preceding sH1N1 shared approximately 70% of the T-cell epitopes[82], such that cross reactivity is the most likely explanation of the strong Teff correlations. In contrast, there was practically no B-cell epitope homology between pH1N1 and sH1N1, which makes Bmem cross reactivity unlikely. A potential explanation of the positive correlation between sH1N1 and pH1N1 antibody titers is that in our study participants the magnitude of the antibody responses to sH1N1 vaccination or infection was likely limited by the same factors that limited the magnitude of the antibody responses to pH1N1 IIV1. Alternatively, sH1N1 and pH1N1 cross-reactive helper T cells may have contributed to the increased antibody responses to pH1N1 in subjects with pre-existing immunity to sH1N1. This latter hypothesis is also in agreement with the association between pH1N1 HAI titers and IFNγ SFC observed in our study.

pH1N1-HAI titers significantly correlated with IFNγ SFC at baseline and post-dose 1, but only marginally at post-dose 2. The baseline and post-dose 1 correlations are in agreement with the Th1-dependence of influenza antibody responses [83,84]. In addition, at entry, 21% of the study population had serologic evidence of past wild-type pH1N1 infection, which probably contributed to the baseline correlation between pH1N1-HAI and Teff, since presumably participants with past infection had higher HAI titers and IFNγ SFC compared with those without past infection. The lack of correlation between pH1N1-Bmem and pH1N1-HAI titers was surprising and contrasted with our previous findings in HIV-infected children and non-pregnant youth. Although the limited number of data points available for these analyses may have also limited the ability to detect the correlations above, this observation needs to be further explored in the context of pregnancy.

This study had several limitations. The use of a single regimen, which included 2 double-doses of pH1N1 IIV1, did not allow precise determination of the effect of increasing the amount of antigen and of the number of doses on the immune response to the vaccine. Studies comparing the immunogenicity of double and standard doses of pH1N1 IIV1 in other HIV-infected populations reported contradictory results[85–87]. The second dose of vaccine in our study did not significantly improve pH1N1-antibody or CMI, which is in agreement with other reports[85]. Our results also have to be interpreted with caution due to the lack of information on correlates of protection against influenza infection and/or disease in HIV-infected individuals.

For the two measures of IIV immunogenicity that are mechanistically associated with protection against influenza infection and disease, antibodies and Teff, our study showed a significant handicap in HIV-infected pregnant women compared with uninfected, nonpregnant historical controls: antibody responses were lower than those of historical controls and IFNγ Teff decreased after multiple doses of vaccine. The decreased immunogenicity of pH1N1 IIV1 was most closely correlated with increased HIV replication and with high proportions of activated and regulatory T cells and exhausted and regulatory B cells. Pregnancy, which is associated with increased frequencies of regulatory T cells, may contribute to the decreased immunogenicity of the vaccine as suggested by recent studies[18,19]. The effects of HIV treatment on the parameters that were associated with low immunogenicity of pH1N1 in this study appear to be as follows: HIV replication can be controlled with appropriate cART, but there is a dearth of information on regulatory B cells in the context of treated or untreated HIV infection and no evidence yet that activated and regulatory T cells and exhausted B cells completely normalize with cART. Until such evidence is presented, special immunization regimens or vaccines with enhanced immunogenicity (e.g., adjuvanted vaccines) need to be considered for optimizing the protection conferred by IIV to HIV-infected pregnant women.

## Supporting Information

S1 Fig Study diagram. (TIFF)

S2 Fig Gating strategy. The figure illustrates a typical example of the gating strategy. Lymphocyte were identified by forward and side scatter. Next, CD4+ T cells were identified as being CD3+CD8- lymphocytes and CD8+ T cells as CD3+CD8+ lymphocytes. Next, the subset of interest was identified by the expression of its characteristic marker. Examples are shown for CD4+TGFb+, CD4+CD39+, CD4+HLADR+CD38+ subsets and their CD8+ counterparts. B cells were identified as CD19+CD3- lymphocytes (not shown).
(TIFF)

S1 Table Phenotypic Characterization of B- and T-Cell Subsets at Baseline and after pH1N1 Vaccination. (TIFF)
