# Supplementary figures and images for: Determinants of Vaccine Immunogenicity in HIV-Infected Pregnant Women: Analysis of B and T Cell Responses to Pandemic H1N1 Monovalent Vaccine
Source: PLoS One. 2015 Apr 13;10(4):e0122431. doi: 10.1371/journal.pone.0122431 (PMC4395240; doi:10.1371/journal.pone.0122431)

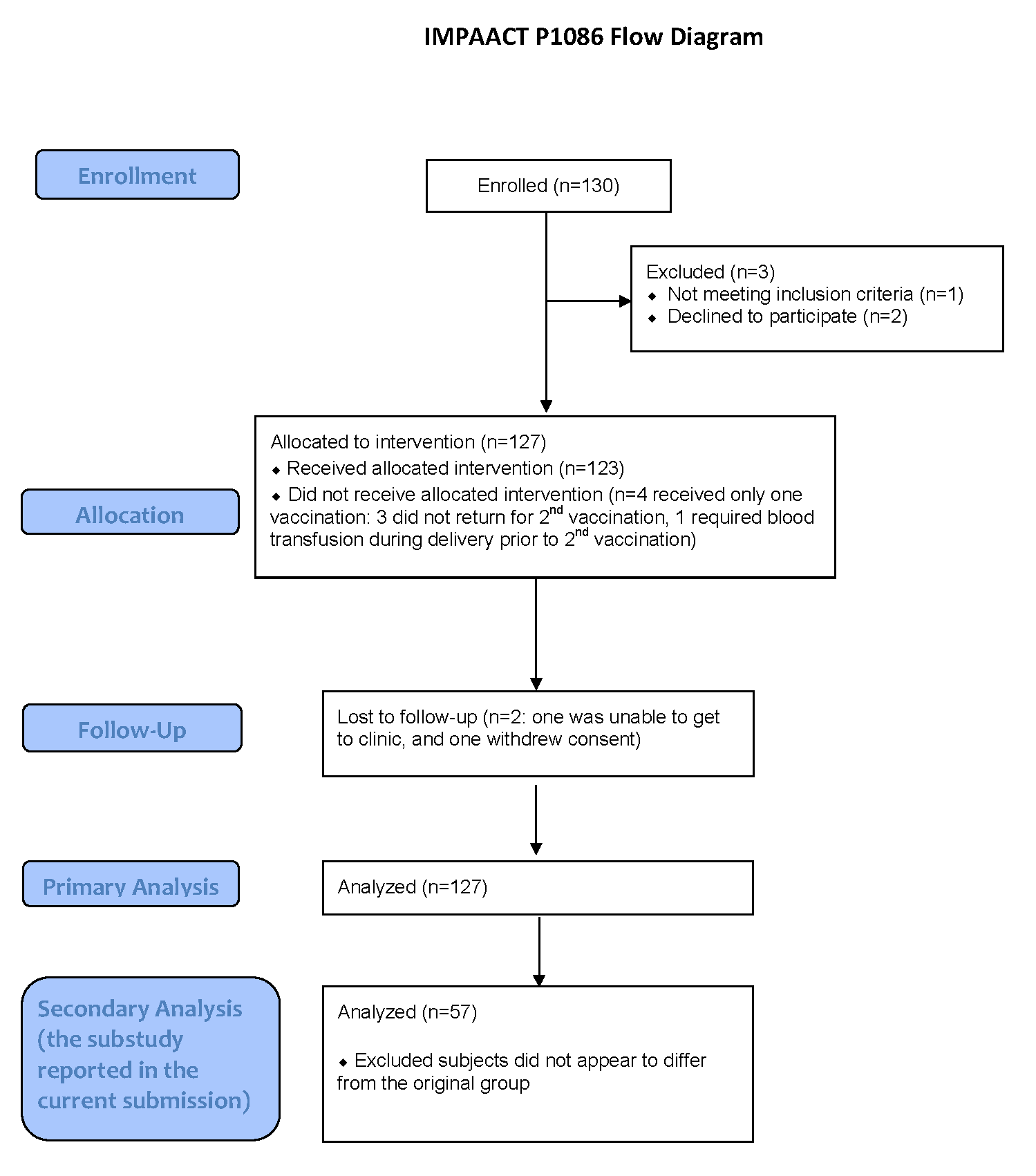

Supplement: S1 Fig — (TIFF) [file pone.0122431.s001.tiff]

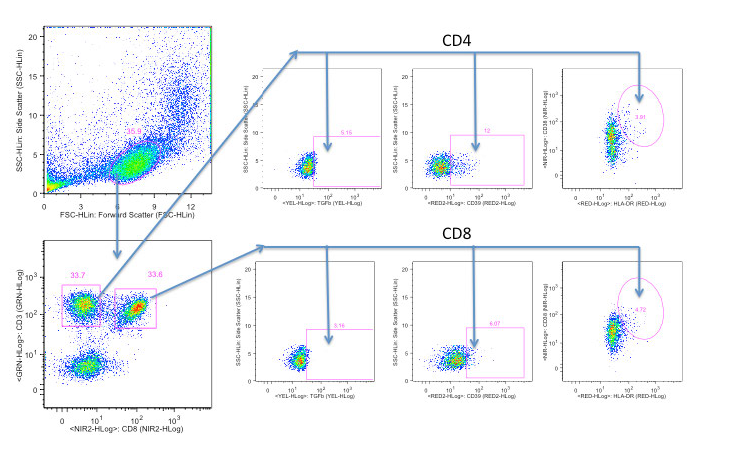

Supplement: S2 Fig — The figure illustrates a typical example of the gating strategy. Lymphocyte were identified by forward and side scatter. Next, CD4+ T cells were identified as being CD3+CD8- lymphocytes and CD8+ T cells as CD3+CD8+ lymphocytes. Next, the subset of interest was identified by the expression of its characteristic marker. Examples are shown for CD4+TGFb+, CD4+CD39+, CD4+HLADR+CD38+ subsets and their CD8+ counterparts. B cells were identified as CD19+CD3- lymphocytes (not shown). (TIFF) [file pone.0122431.s002.tiff]
